# Effects of Chronic Pain Medications on Cardiovascular Health: A Narrative Review

**DOI:** 10.7759/cureus.113644

**Published:** 2026-07-30

**Authors:** Andrew Kent, Ammar Yaser Mohammad Toubasi, Amanda Myles, Anterpreet Dua, Vishal Arora, Yanbin Dong, Zhuo Sun

**Affiliations:** 1 Anesthesiology and Perioperative Medicine, Wellstar Medical Center, Medical College of Georgia, Augusta, USA; 2 Anesthesiology, Boston Medical Center, Boston, USA; 3 Anesthesiology, Piedmont Athens Regional Center, Athens, USA; 4 Cardiology/Interventional Cardiology, Wellstar Medical Center, Medical College of Georgia, Augusta, USA; 5 Georgia Prevention Institute (GPI), Wellstar Medical Center, Medical College of Georgia, Augusta, USA

**Keywords:** acute cerebrovascular accident, cardiovascular risk factors (cvrf), chronic pain management, clinical practice guidelines, multimodal pain management, nsaid medication, opiate agonist, standard of care

## Abstract

Cardiovascular disease (CVD) and chronic pain are leading causes of morbidity and functional disability worldwide, and there is increasing evidence demonstrating that chronic pain may elevate the risk for major adverse cardiac and cerebrovascular events. Given the intertwined nature of the cardiovascular and pain regulatory systems, medications used for chronic pain management may also contribute to the risk of cardiac events due to their cardiovascular side effects. This literature review critically evaluates the impact of the most commonly prescribed pain medications on the cardiovascular system. Medications discussed include non-steroidal anti-inflammatory drugs (NSAIDS), acetaminophen, opioids, antidepressants, gabapentinoids, and intra-articular injections. In each case, factors such as dosage and patient predispositions are considered in how they change a clinician’s response in order to achieve the best patient outcome. A comprehensive literature search was performed on multiple databases, including PubMed, Google Scholar, and ScienceDirect, up to April 2026. For each drug class, cardiovascular risk was evaluated in the context of dosage and patient-specific predisposing factors, and weighed against the degree of pain relief provided. By drawing attention to this issue, our aim is to enhance understanding of drug mechanisms and identify appropriate treatment options, ultimately fostering better clinical practices and evidence-based initiatives in this healthcare endeavor.

## Introduction and background

Pain and cardiovascular (CV) regulation share overlapping neural substrates. Pain-which is distinct from nociception, the neural encoding of noxious stimuli-is a subjective experience shaped by cognitive, emotional, and contextual factors and modulated by the periaqueductal gray and rostral ventromedial medulla, endogenous opioid and descending noradrenergic and serotonergic pathways, and the arterial baroreflex [1,2]. Because the noradrenergic and baroreflex pathways that modulate pain also govern autonomic CV control-the baroreflex regulating both nociceptive sensitivity and, through its efferent output, heart rate and vascular tone-the two systems are physiologically linked before any drug is introduced. This review does not trace where along these pathways individual agents act; the focus is each medication's mechanism and its direct CV effects.

Several terms central to this review warrant explicit definition. Chronic pain is defined here as pain that persists or recurs for longer than three months. This temporal threshold distinguishes it from acute pain and carries pharmacologic implications: chronic pain typically entails sustained, often daily, drug exposure over months to years, so the CV consequences of these medications reflect cumulative and steady-state effects rather than the transient exposure of short-term use. CV risk, as used throughout, refers to the probability of an adverse CV event-principally myocardial infarction (MI), stroke, heart failure, arrhythmia, or CV death-rather than to any single unified endpoint; where the evidence permits, we specify which of these outcomes a given medication most affects, as the risks are not equivalent across drug classes. CV risk factors are the established modifiable and non-modifiable contributors to that probability, including hypertension, dyslipidemia, diabetes mellitus, tobacco use, obesity, advancing age, and pre-existing CV disease (CVD). CV health denotes the absence of clinically significant CVD together with the preservation of normal cardiac and vascular function. This review concerns adults with chronic non-cancer pain, and unless otherwise specified, findings should be understood to apply to that population.

In addition to the modulatory pathways listed above, chronic systemic inflammation, chronic tachycardia, adrenaline release, increased low-density lipoprotein cholesterol, and overlapping risk factors are other pathways of importance. Furthermore, commonly prescribed pain medications such as non-steroidal anti-inflammatory drugs (NSAIDs), analgesics, opioids, gabapentinoids, intra-articular corticosteroids, and antidepressants act on prostaglandin synthesis, autonomic tone, cardiac conduction, and fluid balance, each of which can adversely affect the CV system. According to Chung et al., the overall major adverse CV cerebral event (MACCE) in participants with chronic pain is 27.6%, compared to 19.6% in those without chronic pain, a statistically significant finding [3]. The complex interplay between disease states and the use of preventive and therapeutic medications for them can be challenging to discern and is a substantial and growing clinical challenge. Chronic pain affects more than 100 million adults in America alone, costing the healthcare system approximately $635 billion annually [4,5].

The clinical urgency of this question has increased for demographic and prescribing reasons rather than purely mechanistic ones. Chronic pain and CVD frequently coexist in the same patients, as reflected in the MACCE difference reported above [3]. Yet, several of the trials that anchor the analgesic safety literature deliberately excluded patients with significant CVD, and the studies that have specifically examined older or CV-compromised populations consistently report higher risk estimates than trial averages-patterns documented in detail in the sections that follow. The population in which analgesic CV safety matters most has therefore been the least well represented in the evidence base, and clinicians are frequently required to extrapolate from cohorts healthier than the patients in front of them.

This review focuses on adults with chronic non-cancer pain, with particular attention to those who have established CVD or multiple CV risk factors. Pediatric populations, acute and perioperative pain, and cancer-related pain are outside its scope. The specific question addressed is as follows: among the pharmacologic agents most commonly prescribed for chronic non-cancer pain, what is the current evidence regarding CV risk, and how should that evidence inform prescribing in patients who are already at elevated CV risk?

Six medication groups are examined: NSAIDs, acetaminophen, opioids (including tramadol and the agents used in opioid use disorder), antidepressants (tricyclic antidepressants (TCAs), selective serotonin reuptake inhibitors (SSRIs), and serotonin-norepinephrine reuptake inhibitors), gabapentinoids, and intra-articular injections (IAIs). These groups were selected because they represent the agents most frequently prescribed for chronic non-cancer pain and because each has an established or plausible mechanism by which it may influence CV physiology. A synthesis of this kind is needed because the evidence for these classes has accumulated piecemeal, in separate literatures with differing study designs, comparators, doses, and populations, which makes it difficult for a clinician to weigh one option against another at the point of care.

**Figure 1:**
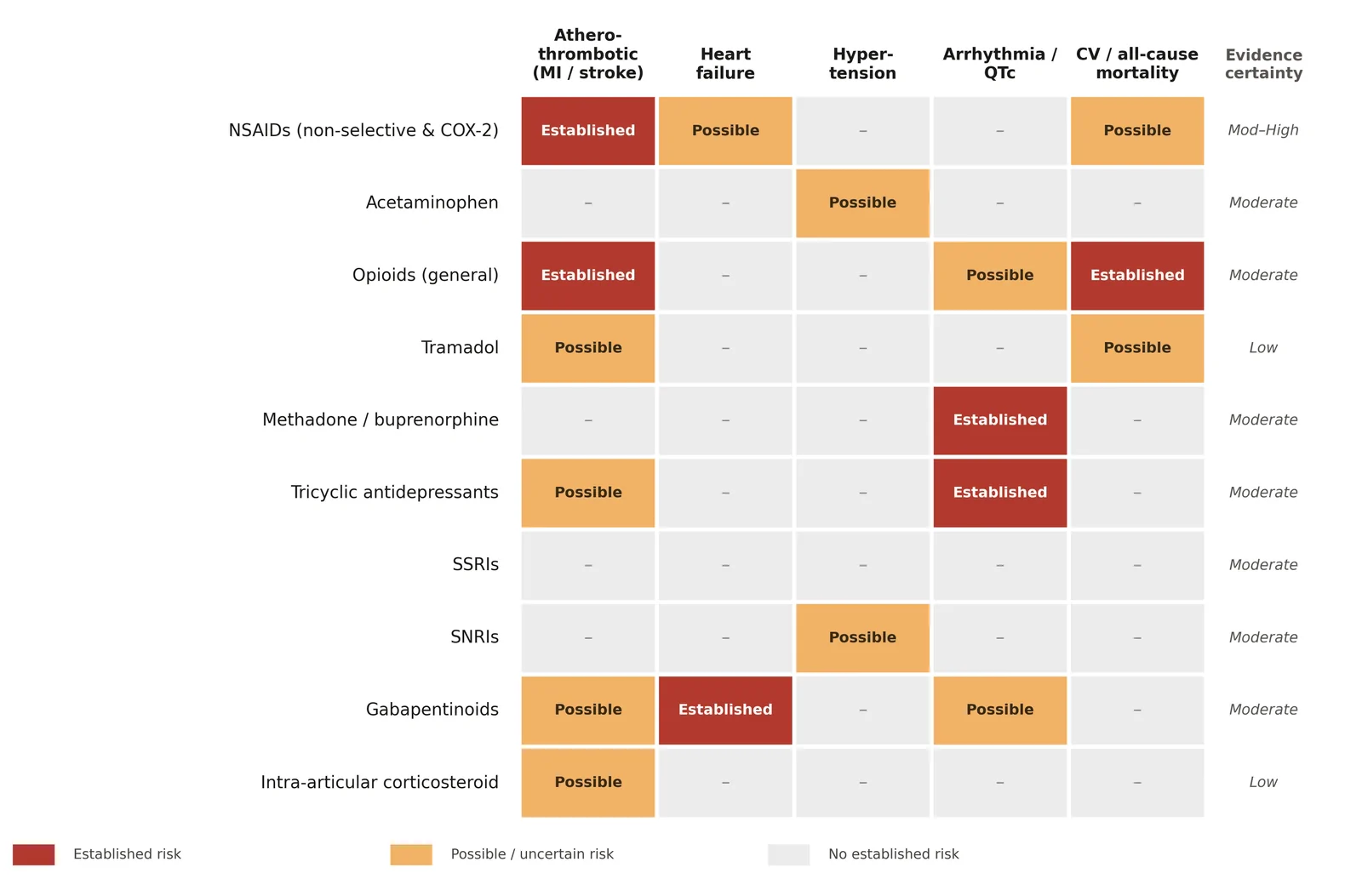
Predominant cardiovascular (CV) risk by drug class and specific CV endpoint. Categories reflect the direction and consistency of evidence summarized in Table 1 and the text of this narrative review, not formally pooled or graded effect estimates. "Established" indicates a consistent signal across multiple studies; "possible or uncertain" indicates a signal of limited magnitude, consistency, or certainty; and a dash indicates no established mechanism or association for that class. Evidence certainty reflects the authors' overall appraisal and is not derived from a formal grading instrument. Intra-articular hyaluronic acid is omitted as it carries no established systemic CV risk. NSAIDs: non-steroidal anti-inflammatory drugs; SSRIs: selective serotonin reuptake inhibitors; SNRIs: serotonin and norepinephrine reuptake inhibitors; MI: myocardial infarction

Ultimately, the goal of this literature review is to address the challenge of managing chronic pain patients with concurrent CVD. By comparing study designs, doses, and populations across drug classes rather than summarizing each in isolation, we hope to improve the understanding of drug mechanisms and appropriate treatment options for these patients, leading to better clinical practice guidelines and evidence-based initiatives focused on this healthcare endeavor. Figure 1 serves as a visual roadmap for the predominant CV risk by drug class and specific CV endpoints.

**Table 1:** Cardiovascular effects of medications commonly used in chronic pain, by drug class. Certainty reflects the authors’ overall appraisal of study design, consistency across studies, and vulnerability to confounding, and is not derived from a formal grading instrument such as GRADE. NSAIDs: non-steroidal anti-inflammatory drugs; RCTs: randomized, placebo-controlled trials; SSRIs: selective serotonin reuptake inhibitors; SNRIs: serotonin and norepinephrine reuptake inhibitors

Drug class	Principal evidence base	Main cardiovascular outcomes	Highest-risk populations	Direction of effect	Certainty
NSAIDs (non-selective and COX-2 selective)	RCTs (CLASS, VIGOR, APPROVe, PRECISION); network meta-analysis; pharmacovigilance analysis	Myocardial infarction, stroke, heart failure, cardiovascular death	Pre-existing cardiovascular disease; higher doses; high-dose COX-2 selective agents	Increased; strongly dose-dependent. Ibuprofen and diclofenac carried the highest stroke risk	Moderate to high
Acetaminophen	Retrospective cohorts; RCT (PATH-BP); prospective cohort; narrative reviews	Systolic blood pressure elevation; myocardial infarction and stroke not significantly increased	Regular high-dose (4 g/day) use in treated hypertension; concurrent antihypertensive therapy	Neutral for myocardial infarction and stroke; modest systolic blood pressure increase with sustained high-dose use	Moderate
Opioids (general)	Prospective cohort (REGARDS); systematic review and meta-analysis; case-control analysis	Cardiovascular death, coronary heart disease, myocardial ischemia, stroke, atrial fibrillation	High dose; prolonged duration; high cumulative prescription burden; concurrent benzodiazepine use	Increased, scaling with dose and duration	Moderate; limited by confounding by indication
Tramadol	Population-based and retrospective cohorts; one animal study	All-cause mortality, myocardial infarction, venous thromboembolism	Pre-existing cardiovascular disease; risk estimates vary substantially by comparator drug	Mixed and comparator-dependent; higher risk versus naproxen, comparable versus codeine or diclofenac	Low
Methadone and buprenorphine	Literature reviews; large veterans cohort	QTc prolongation, torsades de pointes, atrial fibrillation	Baseline QTc >450-500 ms; concurrent QTc-prolonging agents; structural heart disease	Increased arrhythmia risk; more pronounced with methadone	Moderate
Tricyclic antidepressants	Retrospective matched cohorts	Major adverse cardiovascular events, myocardial infarction, stroke, arrhythmia	Pre-existing cardiovascular disease; prolonged use; risk observed even at low dose	Increased	Moderate
SSRIs	Large national database cohorts	Major adverse cardiovascular events, myocardial infarction, arrhythmia, stroke	High-dose users, in whom continued monitoring is advised	Neutral overall, with some evidence of reduced risk in patients with coronary artery disease	Moderate
SNRIs	Meta-analyses of RCTs; drug-specific meta-analysis; retrospective adverse-drug-reaction analysis	Systolic and diastolic blood pressure elevation, heart rate increase, orthostatic hypotension	Venlafaxine at doses above 300 mg/day; older adults with pre-existing cardiovascular disease (duloxetine)	Small average increase in blood pressure and heart rate, but heterogeneous by agent and dose	Moderate
Gabapentinoids	Propensity-score-matched cohorts; Medicare cohort; systematic reviews; case report	Heart failure, myocardial infarction, pulmonary embolism, peripheral vascular disease, atrial fibrillation	Older adults; pre-existing cardiovascular disease or heart failure; longer duration of use	Increased; heart failure signal more pronounced for pregabalin than gabapentin	Moderate
Intra-articular corticosteroids	Case-crossover/cohort study; mixed-methods cohort (RUbICOn)	Acute coronary syndrome	Not clearly differentiated by subgroup in available data	Possible transient acute increase; the largest cohort found numerically higher event rates that did not reach significance	Low
Intra-articular hyaluronic acid	Meta-analysis of RCTs	No significant safety signal identified	Not applicable	Neutral	Moderate

## Review

Evidence acquisition

This article is a narrative review rather than a systematic review. It does not follow the PRISMA 2020 reporting guideline, was not conducted under a registered protocol, and did not employ duplicate independent screening, formal risk-of-bias scoring, or quantitative synthesis. The account below is provided to make the search and selection process as transparent and reproducible as the narrative format allows, and the synthesis should be read as a structured expert overview rather than an exhaustive or formally graded appraisal.

A literature search was performed in PubMed, Google Scholar, and ScienceDirect. The search was first conducted in 2023 and updated on April 15, 2026, with the update repeating the original strategy restricted by publication date; this accounts for the inclusion of studies published between 2024 and 2026. Only English-language publications were considered. Search terms combined the name of each drug class and its principal agents with CV outcome terms and population terms, using the Boolean operators AND and OR. Representative agent terms included “NSAID,” “celecoxib,” “rofecoxib,” “ibuprofen,” “naproxen,” “diclofenac,” “acetaminophen,” “paracetamol,” “opioid,” “tramadol,” “methadone,” “buprenorphine,” “gabapentin,” “pregabalin,” “amitriptyline,” “nortriptyline,” “duloxetine,” “venlafaxine,” and “corticosteroid injection.” Outcome terms included “cardiovascular,” “myocardial infarction,” “stroke,” “heart failure,” “arrhythmia,” “atrial fibrillation,” “QTc,” and “mortality.” Population terms included “chronic pain,” “osteoarthritis,” “rheumatoid arthritis,” “neuropathic pain,” “diabetic neuropathy,” and “fibromyalgia.” The reference lists of included articles and relevant reviews were hand-searched to identify additional studies not captured by the database search.

Eligibility was defined by population, exposure, comparator, and outcome. The population of interest was adults with chronic non-cancer pain, with emphasis on those with established CVD or CV risk factors. The exposures of interest were the pharmacologic classes commonly used for chronic pain: NSAIDs, acetaminophen, opioids (including tramadol and agents used in opioid use disorder), antidepressants (TCAs, SSRIs, and serotonin-norepinephrine reuptake inhibitors), gabapentinoids, and IAIs. Comparators included placebo, no exposure, or an alternative analgesic. Outcomes of interest were CV events, principally MI, stroke, heart failure, arrhythmia, and CV or all-cause mortality.

Primary clinical evidence was drawn from randomized controlled trials, systematic reviews and meta-analyses, and large population-based cohort or case-control studies published within the preceding 25 years. Conference abstracts were not used as sources. Animal studies and case reports were not used as primary clinical evidence; however, a small number were included selectively as supportive mechanistic or illustrative material where direct clinical data were sparse, and are identified as such in the text. Specifically, one murine study is cited to illustrate proposed mechanisms of tramadol cardiotoxicity, for which human mechanistic data are limited, and one case report is cited to illustrate gabapentin-associated atrial fibrillation in an atypically young patient. These sources are presented as hypothesis-generating context and were not weighted as clinical evidence of risk.

Study selection and data extraction were performed by the lead author and reviewed by the senior author; this review was not conducted as a formal independent duplicate process, and this is acknowledged as a limitation. Because the review is narrative, a formal risk-of-bias instrument such as RoB 2, Risk Of Bias In Non-randomized Studies of Exposures (ROBINS-E), or AMSTAR 2 was not applied to individual studies. Instead, the methodological strengths and limitations of key studies-including study design, sample size, adjustment for confounding, and susceptibility to confounding by indication-are appraised qualitatively within the text at the point each study is discussed. Where findings conflicted, greater interpretive weight was given to larger studies, to those with longer follow-up, and to those with more complete adjustment for baseline CV risk, and the basis for that weighting is stated in the relevant section. Studies were grouped by pharmacologic class for synthesis, and cross-cutting themes are consolidated in the Discussion.

Results

Non-steroidal Anti-inflammatory Drugs

NSAIDs are the first-line treatment for pain, working by inhibiting the synthesis of prostaglandins through the inhibition of central cyclooxygenase enzymes. It is important to note that the safety of NSAIDs for CV events in patients with cardiac comorbidities has been examined in meta-analyses and both retrospective and prospective clinical studies. It was found that CV risk varies across the class, with each agent carrying a distinct profile driven largely by its degree of COX-2 selectivity.

Clinical evidence: One such study was performed to examine the safety profiles of celecoxib (400 mg twice/day) against ibuprofen and diclofenac. Referred to as the Celecoxib Long-term Arthritis Safety Study (CLASS) trial, the study found no statistically significant results as each medication produced a similar number of measured CV incidents [6]. Notably, unlike the VIGOR study below, aspirin was used by 20% of patients as a preventative measure, and 27% of participants had rheumatoid arthritis while osteoarthritis drove NSAID use in the remainder. Because the trial was not placebo-controlled and a meaningful minority were concurrent aspirin users, a class-wide CV signal may have been masked; CLASS is therefore best read as evidence of relative rather than absolute CV safety among the agents compared.

Solomon et al. conducted a meta-analysis of six randomized, placebo-controlled trials (RCTs), which included a total of 7,950 patients and 16,070 patient-years of follow-up to investigate the CV risks associated with celecoxib [7]. The group accounted for confounding variables by using Cox proportional hazards models, stratified by study and aspirin use, to handle differences across trials and aspirin use at baseline. They also accounted for baseline CV risk in the study. When comparing placebo to all other dosages, the relative risk (RR) was 1.6. Dosage regimens were examined individually in addition to the above, and the risk was found to be as follows: 400 mg 1x/day-hazard ratio (HR) = 1.1 (95% confidence interval (CI) 0.6-2.0); 200 mg 2x/day-HR = 1.8 (95% CI 1.1-3.1); 400 mg 2x/day-HR = 3.1 (95% CI 1.5-6.1). Pre-existing CV risk and analgesic dose therefore contribute jointly to the overall risk of an unfavorable CV event.

Rofecoxib was evaluated in two trials with concordant results. In the Adenomatous Polyp Prevention on Vioxx (APPROVe) trial, rofecoxib was compared with placebo, and increased occurrence of both MI and stroke was noted: 1.5 occurrences per 100 patient-years with rofecoxib versus 0.78 with placebo (RR = 1.92, P = 0.008) [8]. It took 18 months following the beginning of drug administration before the RR was determined to be significant. The VIGOR study included 8,076 volunteers with rheumatoid arthritis, excluded individuals with active or severe CVD, and disallowed low-dose aspirin, comparing rofecoxib against naproxen [9]. The CV risk ratio of rofecoxib relative to naproxen was 2.37 (95% CI 1.39-4.06; P = 0.0016). Because VIGOR excluded patients with severe CVD while the Trelle et al. meta-analysis below encompassed the full spectrum of baseline risk, the elevated risk with rofecoxib appears to hold regardless of prior CV risk stratification.

Trelle et al. conducted a meta-analysis of 31 total studies, which included 116,429 patients and a corresponding follow-up of 115,000 patient-years [10]. The studies compared multiple NSAIDs (including naproxen, ibuprofen, and diclofenac) and multiple coxibs (including rofecoxib, celecoxib, lumiracoxib, and etoricoxib) and measured their corresponding effect on resulting outcomes including acute MI (AMI), death, death from any cause, and stroke. This monitoring encompassed over 100 patient-years and did not stratify by previous CV risk. Looking at AMI as a primary endpoint, the two analgesics with the highest risk relative to placebo included rofecoxib with an RR of 2.12 (95% CrI 1.26-3.56) and lumiracoxib with an RR of 2.00 (95% CrI 0.71-6.21). Coming in third was the NSAID ibuprofen with an RR of 1.61 (95% CrI 0.50-5.77) relative to placebo. When considering stroke as the primary endpoint measurement, it was found that the non-selective NSAIDs actually promoted the highest risk, with ibuprofen at 3.36 (95% CrI 1.00-11.6) and diclofenac at 2.86 (95% CrI 1.09-8.36).

The PRECISION trial was a multicenter RCT involving 24,081 patients [11]. The study aimed to compare adverse events associated with the daily use of ibuprofen, naproxen, or celecoxib in osteoarthritis patients. When considering CV events as the primary endpoint, celecoxib was found to be non-inferior to both ibuprofen and naproxen with three years of follow-up. Specific findings showed that the rate of CV events was about 2% to 3% in each group. This appears to conflict with VIGOR and with the Trelle et al. meta-analysis, but the discrepancy may reflect a dosing asymmetry rather than a true difference in drug safety: the celecoxib arm used low doses (~200 mg daily) while the naproxen arm used 350 mg twice daily, a relatively medium-to-high dose. A pharmacological critique of both PRECISION and the similarly designed SCOT trial argues that neither study compared celecoxib against equipotent analgesic doses of its NSAID comparators, biasing both trials toward underestimating celecoxib's relative CV risk [12]. In our interpretation, the apparent divergence across these trials may reflect a shift toward progressively lower, more cautious celecoxib dosing in later trial design rather than authentic new reassurance about COX-2 inhibitor safety at the doses historically used in practice.

Observational and pooled data are concordant with these trial findings. NSAID use in patients with previously diagnosed CVD is associated with increased risk of MI, stroke, and death [13]. McGettigan and Henry similarly reported elevated risk of MI, stroke, and heart failure, particularly with COX-2 inhibitors and particularly among patients with pre-existing CVD or multiple risk factors [14]. A large meta-analysis of 639 trials of selective COX-2 inhibitors found a 37% (RR 1.37 (95% CI 1.14-1.66, P = 0.0009)) increase in fatal MI, non-fatal MI, and stroke [15].

A 2024 pharmacovigilance-pharmacodynamics study offers a mechanistic explanation for this gradient. Using the FDA Adverse Event Reporting System (FAERS), the authors examined 13 different NSAIDs and their association with adverse CV events [16]. By looking at over 200,000 adverse drug events and comparing these events to drug binding data using the BindingDB database, the researchers found that higher COX-2 receptor occupancy was positively correlated with cardiac and vascular disorders, providing mechanistic insight into why certain NSAIDs carry greater CV risk.

Clinical implications: Overall, there is evidence indicating that NSAIDs may increase the risk of CV events in patients with chronic pain. The evidence especially points to increased risk associated with COX-2 inhibitors relative to non-selective NSAIDs. However, the degree of risk may vary depending on the type and dosage of NSAID used, as well as individual patient characteristics. Therefore, clinicians should carefully assess the potential CV risks and benefits of NSAID use in patients with chronic pain, especially those with pre-existing CVD or multiple CV risk factors. Considered together, the NSAID literature converges on three points rather than a single verdict. First, CV risk with NSAIDs is dose-dependent more than it is simply drug-dependent; a low-risk NSAID at a high dose can carry more risk than a high-risk NSAID at a low dose, as illustrated by the Solomon et al. and PRECISION data above. Second, risk is meaningfully modified by baseline CV status, which the earlier trials (CLASS, VIGOR) did not stratify for, whereas a Veterans Health Administration/Department of Defense cohort explicitly characterizes risk-factor gradients within NSAID users and could inform more individualized prescribing guidance [17]. Third, non-selective NSAIDs are not a uniformly safer alternative to COX-2 inhibitors: ibuprofen and diclofenac carried some of the highest stroke risk in the Trelle et al. meta-analysis. Clinically, this argues for individualized CV risk stratification and dose minimization over reflexive avoidance of, or reflexive preference for, any single NSAID.

Acetaminophen

Acetaminophen is another widely used drug class for chronic pain management. This medication works by inhibiting the synthesis of prostaglandins in the central nervous system, resulting in analgesic and antipyretic effects. While some studies have suggested that acetaminophen may be safe and effective for the management of chronic pain, others have found an association between its long-term use and increased risk of CV events.

Clinical evidence: Alchin et al. cast doubt on apparent relationships between acetaminophen use and CVD [18]. The authors state that “putative epidemiologic associations of paracetamol use with kidney disease or CVD, hypertension, gastrointestinal disorders, and asthma largely reflect confounding biases and are of doubtful relevance to short-term use (<14 days).”

Fulton et al. aimed to assess the relationship between acetaminophen prescription data and the risk of MI or stroke in hypertensive patients [19]. A retrospective study found that after allowing for potentially confounding variables, there was no increased risk of stroke or MI in patients who used acetaminophen. In one preclinical study, it was found that acetaminophen is a safe drug in the post-MI setting [20]. Additionally, there were no significant cardioprotective effects of the drug shown. Considering long-term use, a prospective study of 5,429 nursing home residents (mean age 86) followed over 18 months found no association between acetaminophen intake and mortality (HR 0.97; 95% CI 0.86-1.10) or MI [21].

However, a 2018 review states that while there are only a few RCTs on the CV effects of acetaminophen, there is evidence suggesting harm outweighs benefit [22]. For example, some evidence points to a strong relationship between acetaminophen use and increased systolic blood pressure (SBP). It is advisable for physicians to bear this in mind when considering chronic use. Similarly, MacIntyre et al. found that regular daily intake of 4 g of acetaminophen increases SBP in individuals with hypertension by about 5 mmHg as compared to the placebo group, and this consequently increases CV risk [23]. However, both the review article and the RCT performed by MacIntryre’s group have pitfalls that weaken the strength of their conclusions. For example, in the review, the group uses a “title only” search for acetaminophen and paracetamol, which introduces an indexing bias, since many insightful papers discuss both of these medications, even if they are not listed in the title of the paper. MacIntyre et al.’s study was a single-center study that allowed the inclusion of individuals on a variety of blood pressure (BP) medicines, with no limitation on the type or dosage. Thus, it is certainly possible that acetaminophen acts as an effect modifier for increased BP when used in conjunction with a particular BP medicine; however, it is unreasonable to conclude that it increases BP and CV risk by itself, especially considering the findings of the aforementioned studies.

Clinical implications: Overall, the available evidence suggests that acetaminophen may be safe for CV health in patients with chronic pain, especially in those who do not use acetaminophen long-term. However, more studies are needed to fully understand the CV effects of acetaminophen, particularly in patients with pre-existing CVD. Clinicians should carefully consider the risks and benefits of acetaminophen use in their patients with chronic pain and CV risk factors. On balance, the acetaminophen literature is reassuring for short-term, standard-dose use, but the two areas of consistent signal, dose-dependent SBP elevation with regular high-dose intake and possible effect modification in patients concurrently taking antihypertensives, both point to dose and duration, rather than acetaminophen exposure per se, as the variables clinicians should track most closely in chronic pain patients.

Opioids

Opioids are commonly prescribed drugs for chronic pain management, although their mechanism of action is not fully understood. It is believed that opioids mimic the endogenous opioid system by increasing sympathetic nervous system activity, decreasing parasympathetic nervous system activity, and causing vasodilation [24]. Opioids also indirectly affect the CV system by causing respiratory depression, which can result in hypoxemia and increased carbon dioxide levels in the blood [22,25]. Current guidelines from the CDC state that multiple factors should be weighed when prescribing opioids, including the potential for increased risk of CV events in prescription opioid users.

Clinical evidence: Chen and Ashburn noted that limited data are available to suggest an association between chronic opioid administration and an elevated risk for cardiac-related adverse events [26]. Yet, it was emphasized that though solid data are lacking, careful patient selection and monitoring are still important in decreasing risks of harm, as opioid administration can be associated with decreased cardiac function when taken together with medications such as benzodiazepines. Resulting dynamics include bradycardia and vasodilation, which may result in edema and hypotension.

Alternatively, the REGARDS trial, which studied 29,000 patients, discovered that prescription opioid use was linked with an elevated risk of CV death and coronary heart disease for treating chronic pain in female patients [27]. The designers of the study employed multiple tactics to ensure validity and generalizability of the study. For example, the authors used a pill count verification model to reduce recall bias in study participants. Moreover, the authors adjusted for confounding variables by employing Cox proportional hazard models, which adjust for traditional CVD risk factors.

A systematic review and meta-analysis was completed in 2025 by Schofield et al., which supported the conclusion of the REGARDS trial [28]. Specifically, they looked at 1.6 million individuals across 17 studies and found that chronic opioid use was associated with an increased risk for CV accident (CVA) (odds ratio (OR): 1.84 (95% CI 1.45-2.35)) and myocardial ischemia (OR: 1.51 (95% CI 1.40-1.63)). The size of the study along with the authors’ use of bias assessment tools such as the ROBINS-E lend strong credibility to the study’s findings of increased risk association between CV events and chronic opioid use.

These findings are reinforced by an independent dose-response analysis showing that both current opioid use and cumulative prescription burden (11 or more prescriptions) were associated with incrementally increased MI risk, with the highest risk concentrated in morphine, meperidine, and polytherapy users [29]. A dose-response gradient replicated across three methodologically distinct designs-a prospective cohort (REGARDS), a large meta-analysis (Schofield et al.), and a prescription-based case-control analysis-strengthens the case for a genuine causal association rather than confounding by indication alone. Because these designs reported different effect measures (RRs, a pooled OR, and MI risk estimates, respectively), the consistency lies in the direction and dose dependence of the association rather than in directly comparable effect magnitudes. That said, residual confounding remains a limitation common to all three: patients who accumulate higher cumulative opioid doses also tend to have more severe underlying pain conditions and greater comorbidity burden, both of which independently raise CV risk.

Tramadol is a synthetic opioid increasingly preferred by physicians to treat chronic pain patients. As direct human mechanistic data are limited, one murine study is included here for mechanistic context. This study sought to characterize the mechanisms of action of tramadol on the CV system by focusing on oxidative stress, inflammation, and cardiac toxicity, as there is very limited literature describing these effects. The authors found that tramadol caused cardiac damage, shown by the increase in lactate dehydrogenase (LDH), troponin I, and creatine phosphokinase-MB (CK-MB) activities in serum samples [30]. A retrospective cohort study following more than 100,000 osteoarthritic patients for one year found that tramadol was associated with a greater risk of adverse events than usual prescribed NSAIDs. These events included venous thromboembolism, hip fractures, and overall mortality [31]. The authors of this study suggested that tramadol use in patients with previously diagnosed CVD could be linked with a higher risk of CV events. A similar finding was demonstrated in a cohort of patients aged 50 years and older with osteoarthritis [32]. This study employed the use of a Cox hazard model to adjust for CVD risk factors. An elevated incidence of mortality over the course of one year was noted when prescription tramadol was compared to usual NSAID prescriptions: a significantly higher rate of mortality over one year compared with common NSAID prescriptions. Moreover, a population-based cohort study found that patients with osteoarthritis who were started on tramadol had a higher six-month risk of MI than those on naproxen and a similar risk to those on diclofenac or codeine [33]. A population-based retrospective cohort study supported these findings in part, showing that short-term use of tramadol was not associated with an increased risk of cardiac events among patients with non-cancer pain when compared with codeine [34].

The apparent inconsistency in the tramadol findings above, increased MI risk versus naproxen, but comparable risk versus codeine or diclofenac, is in our view more readily explained as a comparator effect than as a true discrepancy in tramadol's own safety profile. Naproxen appears comparatively cardioprotective relative to several other analgesics discussed throughout this review, which would make tramadol look riskier when benchmarked against naproxen than when benchmarked against codeine or diclofenac, neither of which carries an established cardioprotective signal. We note that this explanation is inferred from the pattern of comparators across studies rather than tested directly in any single trial.

Methadone and buprenorphine, commonly used opioids for treating opioid use disorder, could elevate the risk of QTc prolongation and torsades de pointes, a life-threatening arrhythmia. This is especially evident in patients who have a baseline increased risk for prolongation. Thus, electrocardiogram (EKG) monitoring should be utilized in these patients [35]. Additionally, risk factors for QTc prolongation include both underlying conditions such as heart disease and the concomitant application of other QTc-prolonging agents such as TCAs and venlafaxine. In patients who are considering methadone as a treatment option, various factors must be weighed, including a history of QTc prolongation or ventricular arrhythmias. In such patients, with the above risk factors, a screening for a baseline prolonged QTc (>450 to 500 ms) is recommended. Patients with a borderline prolonged QTc (450 to 500 ms) should employ the use of an alternate opioid, and methadone is absolutely contraindicated in those with a QTc over 500 ms. Behzadi et al. also supported the suggestion for periodic monitoring of EKG in high-risk patients, including those on opioid maintenance treatment, as methadone has a greater propensity to induce a long QT interval. In addition to the risk of prolonged QT interval induction, a study composed of 850,000 veterans found that atrial fibrillation development increased by 34% in prescription opioid users [36].

Although there are available tools and methods for the identification of opioid use disorder as well as recommendations for safe opioid use for physicians who manage patients with CVD and stroke, a literature review demonstrated that there is an absence of high-quality evidence [37]. This highlights the need for timely research for improving the care for these patients. Considering this, the American Heart Association developed an advisory statement for healthcare professionals and researchers in the setting of CV and brain health to synthesize the current literature, to provide approaches for identifying patients with opioid use disorder, and to address pain management and overdose [37].

In patients with pre-existing CVD, opioids may increase the risk of CV complications and death. The risk of these events is highest in patients prescribed high doses of opioids or using them for longer periods. These findings emphasize the need for cautious prescribing and monitoring of opioid use in patients with chronic pain.

Clinical implications: Taken as a whole, the opioid-CV literature is more internally consistent than the antidepressant or acetaminophen literature discussed elsewhere in this review: multiple independent cohorts, a large-scale meta-analysis, and mechanistic data all converge on an increased risk of arrhythmia, myocardial ischemia, and CV death that scales with dose and duration. The remaining open question is less whether opioids carry CV risk than how much of that risk is attributable to the drug class itself versus the severity of pain, comorbidity burden, and reduced physical activity that lead a patient to be prescribed opioids in the first place, a confounding structure that no observational design used in this literature can fully resolve.

Antidepressants

Depressive symptoms may exacerbate pain intensity and duration, though the direction of the relationship is unclear [38-40]. As such, antidepressants have become a widely used class of medication in the management of chronic pain patients. TCAs, SSRIs, and serotonin and norepinephrine reuptake inhibitors (SNRIs) are subtypes of antidepressants.

Clinical evidence: TCAs act on multiple neurotransmitter pathways and work by blocking the reuptake of serotonin and norepinephrine in the presynaptic terminal, leading to increased concentration of these neurotransmitters in the synaptic cleft. TCAs have been linked with an increased risk of CV events such as stroke, MI, and arrhythmias in chronic pain patients. Jang et al. found that even at low doses, TCAs were associated with major adverse CV events (MACEs) during primary prevention compared with other antidepressants, and prolonged TCA use correlated with higher risk [41]. Another retrospective cohort study of greater than 16,000 matched patients found that low-dose TCA and particularly low-dose nortriptyline was associated with an increased risk of CV adverse events [42]. Considering this recent research, healthcare providers should carefully weigh the risks and benefits of TCA treatment in chronic pain patients, especially those with pre-existing CVD. Close monitoring of CV function is also recommended for patients receiving TCA therapy.

SSRIs function by inhibiting the reuptake of serotonin, increasing its concentration in the synaptic cleft. Through a National Health Insurance Service database cohort study, antidepressants were not associated with the occurrence of MACEs in patients with depression and ischemic heart disease [43]. Yet, the authors recommend continuous careful monitoring of adverse event development in high-dose SSRI users. A cohort study of greater than 230,000 patients revealed that SSRIs did not show a statistically significant association with an increased risk of arrhythmias or stroke in those diagnosed with depression [44]. There was an indication of a reduced risk of MI with SSRIs and particularly fluoxetine and an increased risk with lofepramine. Overall, recent research studies suggest that SSRIs may have no negative impact on the CV system in chronic pain patients and may even have a beneficial effect. In fact, a large cohort study completed in 2025 found that patients with coronary artery disease (CAD) and generalized anxiety disorder (GAD) who used SSRIs had a significantly lower risk of MACE compared with patients who did not use SSRIs [45]. However, it is essential to consider an individual's medical history and condition before prescribing any medication. Further research is required to fully characterize the CV effects of SSRIs in chronic pain patients.

When evaluating the effects of SNRIs, which work by inhibiting the presynaptic uptake of serotonin and norepinephrine in the synaptic cleft and increasing their effect, several recent studies have examined their CV effects in chronic pain patients. A 2017 meta-analysis of 23 RCTs found that SSRIs did not affect BP, while SNRIs led to a slight elevation in SBP and DBP with statistical significance compared with SSRIs [46]. Current research suggests that SNRI drugs are generally safe for use in chronic pain patients, as they only cause a slight increase in heart rate and/or BP.

More granular, drug-specific data complicate this reassuring class-level summary. A dedicated meta-analysis of duloxetine found statistically significant increases in heart rate (+2.22 beats/min (95% CI 1.53-2.91)) and diastolic BP (DBP) (+0.82 mmHg (95% CI 0.17-1.47)) relative to comparator, and a separate retrospective adverse-drug-reaction analysis in adults aged 70 to 79 with pre-existing CVD identified duloxetine-associated orthostatic hypotension and peripheral vasoconstriction severe enough to warrant dose reduction or discontinuation [47,48]. Venlafaxine appears to carry the strongest and most dose-dependent signal within the SNRI class, with a sustained-hypertension incidence of 4.8% versus 2.1% on placebo, concentrated at doses above 300 mg/day, though case reports of marked BP elevation exist even at 150 mg/day [49]. Taken together, this suggests the reassuring average effect size reported at the class level obscures clinically important heterogeneity, both between individual SNRIs, with venlafaxine appearing riskier than duloxetine, and within a single drug depending on dose and the CV reserve of the population studied. For chronic pain patients specifically, many of whom are older and carry overlapping CV comorbidity, the older-adult-specific data described here are arguably more clinically relevant than the average effect size reported across a general psychiatric population.

A systematic review sought to clarify the adverse effect profiles and tolerability of antidepressants including TCA, SSRI, and SNRI for treatment of chronic pain [50]. However, no specific CV effect of these drugs was reported. Of note, another review stated that as there is “no robust clinical guideline yet, patients should be individually evaluated with respect to their potential risks and benefits from antidepressant therapy” [51]. Thus, to appropriately treat patients with antidepressants to decrease any risk for CVD, the authors recommend an EKG before and after initiating treatment in addition to intermittent EKGs during the treatment course. The authors also recommend obtaining baseline laboratory studies prior to initiation of antidepressant therapy.

Clinical implications: Across the three antidepressant subclasses, a consistent pattern emerges: CV risk tracks with the degree of noradrenergic activity (TCA greater than SNRI greater than SSRI), which follows directly from the strength of each class's reuptake inhibition mechanism rather than appearing as an independent, mechanistically unexplained finding. This gradient is our synthesis across separate literatures rather than a finding of any individual study, and while the mechanistic coherence is suggestive of a real pharmacologic effect, it does not by itself exclude confounding, and it argues for preferential use of SSRIs in chronic pain patients with significant CV comorbidity when efficacy for the specific pain condition is otherwise comparable.

Gabapentinoids

Gabapentinoids, namely, gabapentin and pregabalin, exert their pharmacological action by targeting the alpha-2-delta subunit of voltage-gated calcium channels, thereby decreasing calcium influx and inhibiting the release of certain neurotransmitters such as glutamate and substance P. In terms of the mechanism of gabapentin on hemodynamics, Chen et al. were the first to demonstrate that gabapentin regulates central CV depressor effects via the nucleus tractus solitarii through nitric oxide synthase signaling [52]. This insight can be useful in further understanding and treatment for CVDs. Moreover, in an RCT, the preliminary results indicate that pregabalin may regulate CV and subjective responses to exercise in many fibromyalgia patients [53].

Clinical evidence: Zaccara et al. performed a meta-analysis and found that 51% of adverse events were associated with pregabalin, including peripheral edema [54]. This edema could have been associated with heart failure. However, no significant association between pregabalin and serious adverse events was identified. A larger and more recent systematic review pooling five cohort studies and over one million patients found that gabapentin use was associated with an increased risk of MI after one year of use (HR 1.31, 95% CI 1.14-1.52), along with elevated risk of peripheral vascular disease at both one- and five-year follow-up, adding weight to the signal beyond the single studies summarized above [55].

While the previous study suggested pregabalin can cause edema and possibly heart failure, a subsequent study examined this potential risk of heart failure specifically in older adults receiving pregabalin compared to gabapentin. In the primary analysis, there is no difference in the risk of heart failure with pregabalin compared to gabapentin, and in the secondary analysis, which was stratified for baseline history of heart failure, there were similar findings [56].

This finding is directly contradicted by a subsequently published, considerably larger Medicare claims-based retrospective cohort study, which found that initiating pregabalin (versus gabapentin) was associated with a significantly increased risk of incident heart failure in older adults (adjusted HR 1.48 (95% CI 1.19-1.77)), with the risk rising further in patients with pre-existing CVD (adjusted HR 1.85 (95% CI 1.38-2.47)) [57]. The discrepancy between these two studies is instructive rather than merely contradictory: the newer study used a substantially larger, nationally representative Medicare population with longer follow-up and finer control for baseline heart failure status, and an accompanying editorial characterized it as a well-designed study addressing a genuine area of clinical uncertainty. We interpret this to suggest the comparative CV safety of pregabalin versus gabapentin specifically, as distinct from gabapentinoids as a class versus non-use, was likely underpowered or inadequately captured in the earlier analysis, although the two studies differ in population and design in ways that preclude a definitive comparison and clinicians should weigh the larger, more recent dataset more heavily pending further confirmatory studies.

However, in a cohort study of greater than 210,000 patients who had a diagnosis of diabetic neuropathy, it was found that there was an increased risk for heart failure, MI, and peripheral vascular disease with long-term use [58]. It is well known that diabetes is linked to endothelial damage and accelerated atherosclerosis. Thus, in patients with diabetic neuropathy, an increased risk of CV events is already present, consistent with the expected finding of increased CV risk in this population. However, the authors of this article do note that study participants were stratified based on the number of medications used for diabetic neuropathy in both the gabapentinoid and non-gabapentinoid groups. It is doubtful whether or not medication quantity is an appropriate measure of neuropathic severity. Thus, it is possible that significantly more severe cases of diabetic neuropathy were included in the exposure group when compared to the group that did not receive gabapentinoids. When considering this, the authors of this paper support the conclusion that gabapentinoids can increase the risk of CV events in patients with diabetic neuropathy; however, the degree of risk is still uncertain due to the stratification methods employed in this study.

While the above study included patients with diabetic neuropathy, potentially muddying the waters regarding CV effects that are due to diabetes alone versus due to gabapentinoid usage, a large propensity-score-matched-cohort study performed in 2024 supports the previous study’s findings without this potential confounder. Specifically, the study included 105,602 fibromyalgia patients from 64 US healthcare organizations, which found gabapentin and pregabalin increased risks of multiple CV events at both one- and five-year follow-up [59]. The most notable include MI (HR 1.31, 95% CI 1.03-1.66) and pulmonary embolism (HR 2.23, 95% CI 1.62-3.07). Considering the ubiquity of gabapentinoid usage across the healthcare system, these results are clinically significant and warrant further investigation. The neuropathic benefits of gabapentinoids must be weighed against the increased risk of CV events, and the authors of this article believe that frequently the benefit will not outweigh the risks.

Another concern is the unique side effects of gabapentin, including atrial fibrillation. Studies have shown that gabapentin can be associated with an increased incidence of atrial fibrillation, yet this is usually in patients of at least 65 years of age with comorbidities that predispose to the development of this arrhythmia [60]. Illustratively, and while case reports cannot establish risk, one report described new-onset atrial fibrillation induced by gabapentin in a 20-year-old male patient [61].

Clinical implications: The use of gabapentinoids in chronic pain patients may increase the risk of CV events such as atrial fibrillation and heart failure, especially at high doses and in older patients. Synthesizing this section, the gabapentinoid CV literature has moved in the past two years from single-study signals with acknowledged confounding (diabetic neuropathy severity, medication-count-based severity stratification) toward larger, better-matched cohorts that increasingly implicate fluid retention and possible direct cardiac effects as a real, dose- and duration-dependent risk, one that now appears more pronounced with pregabalin than gabapentin in the oldest and most CV vulnerable patients.

Intra-articular Injections

Intra-articular corticosteroids exert their therapeutic effects by directly interacting with nuclear receptors and impeding the inflammatory and immune cascades at multiple levels.

Clinical evidence: Thomas and Schonmann found a substantial increase in the risk of acute coronary syndrome (ACS) in the week following corticosteroid injection (intra-articular corticosteroid injection (IACI)) (227 versus 31 events per 100,000 visits, OR: 7.3; 95% CI 2.8 to 19.1) [62]. The authors note that the effect size could be clinically significant despite the inappreciable size of the absolute risk. It is important to note that when the analysis was confined to subgroups defined by age, CV risk factors, and sex, the association between receiving IACI and ACS remains similar. The evidence concerning the association between IACI and CV events in chronic pain patients is poor. Based on the evidence reviewed, there may be a clinically significant acute increase in ACS following IACI as well as an increased risk of reported serious adverse events. Nonetheless, further research is required to establish the CV safety of IACI in chronic pain patients.

IAI with hyaluronic acid (IAHA) is commonly used in the treatment of arthritic pain. Honvo et al. assessed the safety profile of IAHA in patients with arthritis through a comprehensive meta-analysis of nine RCTs [63]. They found that IAHA was not associated with significant safety concerns in the management of arthritis.

Lastly, a study completed in 2022 used a mixed-methods model (RUbICOn) analyzing 23,899 osteoarthritis patients who received IACIs. The results of this study were mixed. Specifically, diabetes, ischemic heart disease, and MI occurred more frequently in the injection group, but these differences did not reach statistical significance [64]. Given the event counts involved, the study may have been underpowered to detect a difference of the observed magnitude, so this should not be interpreted as evidence of no effect. While the credibility of this study is strong due to propensity score matching and long-term follow-up, the results leave much to be desired in terms of correlation between cardiac events and intra-articular steroids. Consequently, the authors support shared decision-making between patients and clinicians regarding the risks and benefits of IACI, as the decision to use will be on a case-by-case basis. A complete summary of IAI and the agents above can be found in Table 1.

Clinical implications: Of the five drug classes reviewed here, IACI carries the least mature evidence base: a single case-crossover study drives most of the acute-risk signal, and the largest and most recent cohort (RUbICOn) failed to reach statistical significance despite numerically higher event rates in the injection group. This is a meaningfully different evidentiary situation from the opioid or gabapentinoid literature discussed above, where multiple, methodologically distinct large cohorts converge on the same direction of effect, and it should temper how confidently any CV risk claim is made about IACI relative to the other drug classes in this review.

Discussion

Reviewing the CV safety literature across NSAIDs, acetaminophen, opioids, antidepressants, gabapentinoids, and IAIs reveals several cross-cutting patterns that are easy to miss when each drug class is considered in isolation. Four themes recur with enough consistency to warrant emphasis: the dose and duration dependence of risk, the disproportionate vulnerability of older and CV-compromised patients, the pervasive threat of confounding by indication, and the rapid evolution of the evidence base itself. Considered together, these themes argue against a fixed hierarchy of safe versus unsafe analgesics and in favor of individualized, continually re-evaluated prescribing.

First, in every class reviewed here, the risk signal is graded. The Solomon et al. dose-stratified celecoxib data (RR climbing from 1.1 at 400 mg once daily to 3.1 at 400 mg twice daily), the venlafaxine hypertension signal concentrated above 300 mg/day, the one-year threshold before gabapentin's MI risk became apparent, and the cumulative-prescription gradient in the opioid literature all point to the same conclusion: the meaningful clinical variable is the specific dose and duration of exposure, not merely whether a drug has been prescribed. This has a direct practical corollary, namely, that the lowest effective dose for the shortest necessary duration is likely to mitigate risk across all five classes, and that a low-risk agent used aggressively may be more dangerous than a higher-risk agent used conservatively.

Second, the populations at greatest risk are precisely those most likely to present for chronic pain management. Older adults and patients with pre-existing CVD were disproportionately represented among the highest-risk subgroups for nearly every class discussed, most starkly in the gabapentinoid heart failure data, where the adjusted HR for incident heart failure with pregabalin rose from 1.48 in the general older-adult population to 1.85 in those with established CVD. Because chronic pain, advancing age, and CV comorbidity cluster together in the same patients, population-level average risk estimates almost certainly understate the risk faced by the typical patient a clinician is actually deciding about. Trial and cohort averages drawn from younger or healthier populations should therefore be applied to elderly, comorbid chronic pain patients only with considerable caution.

Third, most of the evidence reviewed here is observational and shares a common structural vulnerability: confounding by indication. Patients prescribed opioids, gabapentinoids, or higher-dose NSAIDs for chronic pain tend to have more severe underlying disease, greater comorbidity burden, and lower physical activity than their comparators, and each of these independently raises CV risk. Even the strongest studies cited here, several of which employed propensity score matching or bias assessment tools such as ROBINS-E, cannot fully eliminate this problem, because the severity of pain that drives the prescribing decision is itself a CV risk factor that is difficult to measure and adjust for. This is why the mechanistically coherent findings in this review, such as the graded TCA-greater-than-SNRI-greater-than-SSRI pattern that tracks noradrenergic activity, or the correlation between COX-2 receptor occupancy and vascular events, carry particular weight: a plausible biological mechanism helps distinguish a genuine pharmacologic effect from an artifact of who receives the drug.

Fourth, this is a genuinely moving evidence base. Several of the most methodologically rigorous studies cited here were published within the past one to two years, and in at least two instances, they directly reframe or overturn earlier work: the PRECISION and SCOT trials appeared to reassure on celecoxib safety until it was recognized that they had not compared equipotent doses, and a large Medicare cohort found a pregabalin-associated heart failure risk that a smaller earlier comparison had missed. The consistent direction of these revisions, toward detecting risk that smaller or earlier studies were underpowered to see, suggests that where the current literature is equivocal, clinicians should lean toward caution rather than reassurance, and should preferentially weight the largest, most recent, and best-controlled datasets.

Taken together, these observations support a unifying clinical stance rather than a ranked list of drugs. For a given patient, the safest choice depends on that individual's baseline CV risk, the specific agent and dose contemplated, the anticipated duration of therapy, and the availability of a mechanistically lower-risk alternative of comparable efficacy for the specific pain condition, for example, favoring an SSRI over an SNRI or TCA, or a non-selective NSAID at a modest dose over a high-dose COX-2 inhibitor, when the clinical situation allows. Equally important, because untreated chronic pain itself carries CV consequences, the goal is not simply to minimize medication exposure but to optimize the net balance between the harms of the drug and the harms of inadequately treated pain. Realizing this balance will require better prospective, adequately powered studies in exactly the elderly, comorbid populations most often excluded from the trials that currently anchor the evidence.

## Conclusions

This narrative review synthesizes current evidence on the CV effects of the medications most commonly prescribed for chronic non-cancer pain. A recurring theme across all six drug classes is that CV risk is graded by dose and duration rather than being an all-or-nothing property of a given drug, and that it concentrates in older patients and those with pre-existing CVD. Critically, "CV risk" is not a single endpoint: the drug classes reviewed here act through distinct mechanisms and threaten distinct outcomes, so risk stratification and monitoring should be directed at the specific hazard each class poses rather than at CV function in general. The predominant hazards differ by class. NSAIDs-particularly COX-2-selective agents at higher doses-carry principally atherothrombotic (MI, stroke) and heart failure risk, and are best avoided or minimized in patients with established atherosclerotic disease or heart failure. Opioids are associated with dose- and duration-dependent increases in myocardial ischemia, CV death, and atrial fibrillation, while methadone and, to a lesser degree, buprenorphine additionally prolong the QTc interval and warrant baseline and follow-up EKG in patients with other QTc risk factors. Gabapentinoids raise the risk of heart failure and peripheral edema, more so for pregabalin than gabapentin, favoring caution and weight and volume monitoring in patients with reduced cardiac reserve. TCAs carry arrhythmic and ischemic risk even at low doses and are the antidepressant class of greatest concern in patients with CVD. By contrast, SSRIs appear CV-neutral or possibly protective; SNRIs are generally safe but can raise BP, most notably venlafaxine at higher doses, warranting BP monitoring. Acetaminophen is generally safe at recommended doses, with a modest dose-dependent rise in SBP during sustained high-dose use. The evidence for tramadol is mixed and of limited quality. None of the reviewed agents carries an established valvular risk.

Because untreated chronic pain itself carries CV consequences, the goal is not to minimize medication exposure per se but to match each patient to the analgesic whose specific CV hazard is least relevant to their baseline profile-selecting an SSRI over a tricyclic in a patient with coronary disease, avoiding COX-2 inhibitors in one with heart failure, or obtaining an EKG before initiating methadone in one with conduction risk. These principles are summarized by class in Table 1. Realizing them in practice requires system-level support: guideline-based pain management pathways that account for CV comorbidity, consistent documentation of analgesic plans beyond the discharge summary, and shared decision-making that weighs analgesic benefit against the specific CV risk at hand. Prospective studies enrolling the older, CV comorbid patients most often excluded from existing trials are needed to refine these recommendations.
